# Prognostic significance of FAM83D gene expression across human cancer types

**DOI:** 10.18632/oncotarget.6620

**Published:** 2015-12-15

**Authors:** Peter J. Walian, Bo Hang, Jian-Hua Mao

**Affiliations:** ^1^ Life Sciences Division, Lawrence Berkeley National Laboratory, Berkeley, CA 94127, USA

**Keywords:** FAM83D, prognosis, human cancer, genetic instability

## Abstract

The family with sequence similarity 83, member D (*FAM83D*) gene has been proposed as a new prognostic marker for breast cancer. Here we further evaluate the prognostic significance of *FAM83D* expression in different breast cancer subtypes using a meta-analysis. Patients with higher *FAM83D* mRNA levels have significantly decreased overall and metastatic relapse-free survival, particularly in the group of patients with ER-positive, or luminal subtype tumors. We also assessed *FAM83D* alterations and its prognostic significance across 22 human cancer types using The Cancer Genome Atlas (TCGA). *FAM83D* is frequently gained in the majority of human cancer types, resulting in the elevated expression of *FAM83D*. Higher levels of *FAM83D* mRNA expression are significantly associated with decreased overall survival in several cancer types. Finally, we demonstrate that *TP53* mutation in human cancers is coupled to a significant increase in the expression of *FAM83D*, and that a higher level of *FAM83D* expression is positively correlated with an increase in genome instability in many cancer types. These results identify *FAM83D* as a potential novel oncogene across multiple human cancer types.

## INTRODUCTION

Cancer is a complex and intrinsically heterogeneous disease in which patients may exhibit similar symptoms, and appear to have the same disease, for entirely different genetic reasons [1, 2, 3]. Microarray and next generation sequencing technologies have been invaluable tools for deconvoluting the heterogeneity and complexity of somatic cancer genetics. These technologies are also facilitating development of a catalogue of genomic changes with which to identify new biomarkers for the diagnosis, prognosis, and prediction of therapeutic response, and the discovery of new therapeutic targets. However, while improvements have been made in the diagnosis and treatment of some cancers, the prognosis and survival for most patients, especially those with metastasis, have not dramatically changed. Therefore, an urgent need exists for new cellular and mechanistic insights into why tumor metastases occur, and for the development of new therapies to improve patient survival and overall quality of life.

The family with sequence similarity 83, member D (*FAM83D*) gene is located on chromosome 20q, a region that is frequently amplified in various types of human cancer. We recently identified *FAM83D* as a novel oncogene for breast cancer (BC) by demonstrating that higher *FAM83D* expression is significantly correlated with shorter disease- and distant metastasis-free surivival in BC patients, and that forced expression of *FAM83D in vitro* promotes BC cell proliferation, migration and invasion, while *FAM83D* depletion by shRNA leads to cell death [4]. *FAM83D* excutes these biological functions at least in part through regulation of the tumor suppressor gene *FBXW7* [4]. The fact that *FAM83D* expression is elevated in hepatocellular carcinoma [5, 6], ovarian cancer [7] and metastatic lung adenocarcinomas [8] suggests the possibility that *FAM83D* is an oncogene for additional cancer types. In this study, using recently available cancer genomic data from ‘The Cancer Genome Atlas' (TCGA), we investigated the prognostic significance of *FAM83D* expression across 22 human cancer types.

Genetic instability is a characteristic of most human cancers and is believed to enable acquisition of other cancer hallmarks [9]. Genetic instability in tumors has been characterized at the level of single nucleotide (mutation), small microsatellite sequences and whole chromosomes (aneuploidy) [10, 11]. Although the mechanisms that control small scale changes (mismatch repair mechanisms) [12, 13, 14] and large scale chromosomal changes [10, 11] have been widely studied, much less is known about the mechanisms that control structural defects, deletions, and amplifications. FAM83D interacts with the chromokinesin KID22 and is required for correct chromosome congression in metaphase [15]. In this study, we utilized the TCGA database to assess whether overexpression of *FAM83D* correlates with genetic instability (e.g. the fraction of cancer genomes with copy number alteration and mutation frequencies).

## RESULT

### *FAM83D* expression and overall and metastatic relapse-free survival in breast cancers

**Figure 1:**
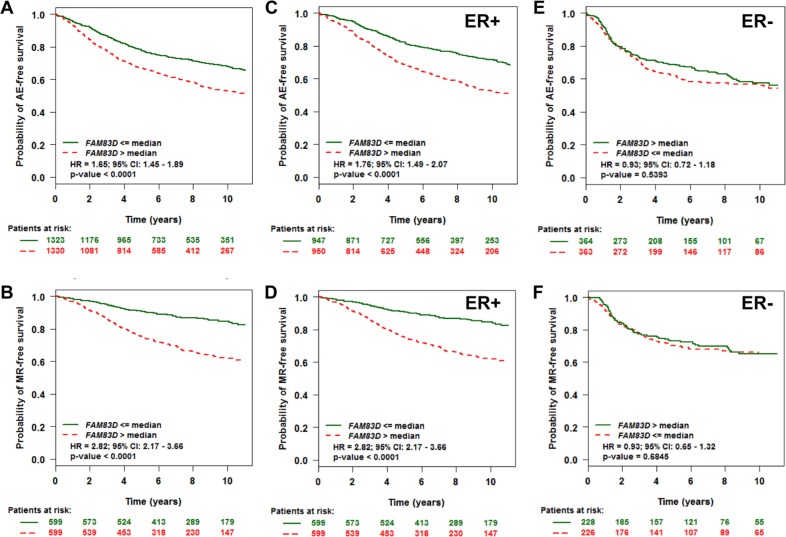
Evaluation of the prognostic impact of *FAM83D* mRNA expression on any event (AE) - and metastatic relapse (MR)-free survival **A.** Association of *FAM83D* expression with AE-free survival. **B.** Association of *FAM83D* expression with MR-free survival. **C–F.** Effect of *FAM83D* expression levels on AE- and MR-free survival according to ER status. “Patients at risk” refers to patients that are at risk of the event occurrence, such as death or metastatic relapse. Kaplan-Meier survival curves for breast cancer patients according to tumor expression of *FAM83D* are presented. The *p* values were obtained from a log-rank test among two groups.

We conducted a meta-analysis of the prognostic significance of *FAM83D* expression in human BC patients using the Breast Cancer Gene-Expression Miner v3.1 (bc-GenExMiner v3.1) software program [16]. Consistent with a previous report [4], patients with high *FAM83D* mRNA expression levels (greater than median expression) had significantly decreased overall survival (OS, any event [AE]) in comparison to those with low *FAM83D* mRNA levels (less than median expression) (Figure 1A, Supplementary Figure S1A). Additionally, high levels of *FAM83D* mRNA were significantly correlated with decreased metastatic relapse (MR)-free survival (MRFS) (Figure 1B, Supplementary Figure S1B). These results indicate that *FAM83D* is a statistically significant biomarker for OS and MRFS.

### *FAM83D* is an independent marker of disease outcome in ER-positive patients

Estrogen receptor (ER) and nodal status in BC are important predictors of recurrence and greatly influence treatment regimens. We therefore performed univariate Cox proportional hazards model analysis on each of the 18 possible pools corresponding to every combination of population (nodal and ER status) and event criteria (MR or any event [AE]) to assess the prognostic impact of *FAM83D* expression on patients with different ER and nodal statuses. As summarized in Table 1, we found that high *FAM83D* expression levels shortened both AE- and MR-free survival only within the groups of ER-positive (ER^+^) or mixed (ER^m^) patients, and not within the group of ER-negative (ER^−^) patients. To further clarify these results, we performed a subset analysis of *FAM83D* in ER+ and ER- tumors. High levels of *FAM83D* expression were significantly associated with shorter AE- and MR-free survival in patients with ER+, but not ER- tumors (Figure 1C–1F).

**Table 1:** Prognostic impact of *FAM83D* expression level in 18 possible pools corresponding to every combination of populations (nodal and ER status)

LN	ER	Event status	*p*-value	Hazard ratio	95% CI	No patients	No events
Nm	**Erm**	AE	**< 0.0001**	**1.34**	**1.26 - 1.43**	2653	909
Nm	**ER+**	MR	**< 0.0001**	**1.56**	**1.41 - 1.71**	1198	282
Nm	**ERm**	MR	**< 0.0001**	**1.44**	**1.33 - 1.56**	1672	425
Nm	**ER+**	AE	**< 0.0001**	**1.42**	**1.31 - 1.54**	1897	624
N+	**ERm**	MR	**< 0.0001**	**1.56**	**1.36 - 1.80**	439	166
N+	**ER+**	MR	**< 0.0001**	**1.63**	**1.38 - 1.92**	343	118
N-	**ERm**	AE	**< 0.0001**	**1.38**	**1.23 - 1.55**	1048	299
N-	**ER+**	AE	**< 0.0001**	**1.5**	**1.29 - 1.75**	743	213
N+	**ERm**	AE	**< 0.0001**	**1.29**	**1.17 - 1.43**	911	406
N-	**ER+**	MR	**< 0.0001**	**1.78**	**1.41 - 2.27**	422	78
N+	**ER+**	AE	**< 0.0001**	**1.33**	**1.18 - 1.51**	685	284
N-	**ERm**	MR	**0.0001**	**1.42**	**1.20 - 1.69**	590	111
Nm	ER-	MR	0.0681	1.17	0.99 - 1.38	454	140
Nm	ER-	AE	0.0878	1.12	0.98 - 1.27	727	278
N-	ER-	AE	0.0909	1.23	0.97 - 1.56	285	82
N+	ER-	MR	0.3033	1.16	0.87 - 1.55	94	48
N-	ER-	MR	0.7622	1.06	0.74 - 1.51	153	32
N+	ER-	AE	0.7638	1.03	0.84 - 1.27	223	122

Node or ER status (+: positive, −: negative, m: mixed); AE: any event; MR: metastatic relapse; HR: hazards ratio.

### *FAM83D* is an independent marker of disease outcome in luminal patients

**Figure 2:**
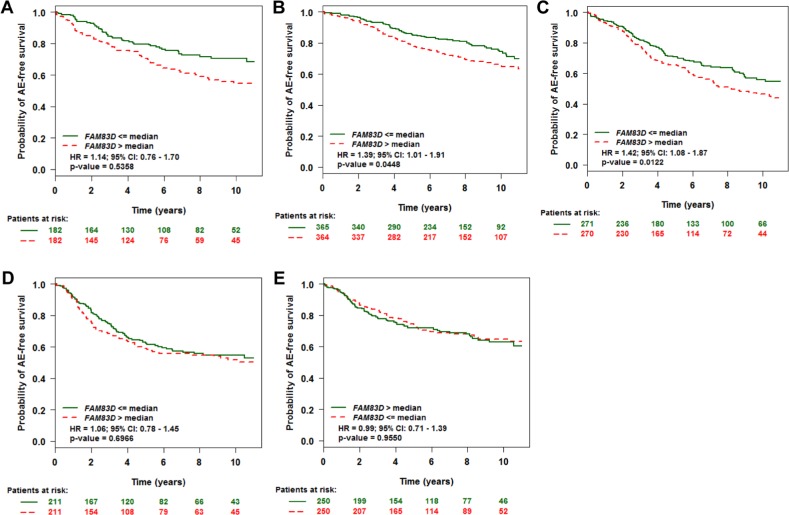
Effect of *FAM83D* expression levels on any event (AE)-free survival according to molecular subtypes **A.** Normal-like, **B.** Luminal A, **C.** Luminal B, **D.** ERBB2, and **E.** Basal subtype. “Patients at risk” refers to patients that are at risk of the event occurrence, such as death or metastatic relapse. Kaplan-Meier estimates of AE-free survival according to the *FAM83D* expression are presented. The *p* values were obtained from a log-rank test among two groups.

We investigated whether *FAM83D* could predict disease outcome within the individual molecular subtypes. Tumors were classified into normal-like, luminal A, luminal B, HER2^+^, and basal-like subtypes based on criteria described by PAM50 [17]. This resulted in samples assigned as normal-like (*n* = 364), luminal A (*n* = 729), luminal B (*n* = 541), HER2^+^ (*n* = 422), or basal-like (*n* = 500). Overall, expression levels of *FAM83D* were relatively high in luminal B, HER2^+^ and basal-like tumors, and low in luminal A and normal-like tumors (Supplementary Figure S2). In luminal A and B subtypes, patients with high expression levels of *FAM83D* presented with significantly decreased AE-free survival (Figure 2B and 2C). Conversely, there was no significant effect of *FAM83D* expression levels on AE-free survival in the normal-like, HER2^+^ and basal-like subtype groups (Figure 2A, 2D and 2E).

### *FAM83D* is an independent marker of disease outcome in several human cancer types

**Figure 3:**
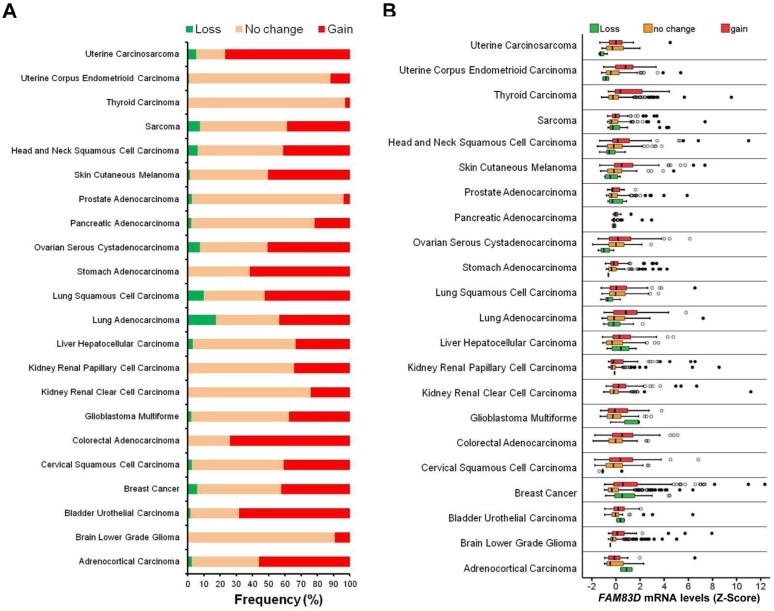
Alteration of *FAM83D* across human cancer types **A.** Frequencies of *FAM83D* genomic alterations in different human cancer types. **B.** Relationship between *FAM83D* mRNA expression levels and genomic alterations.

TCGA data was analyzed to determine whether *FAM83D* might be involved in other cancers (Supplementary Table S1). We found that *FAM83D* was gained in more than 20% of cases in 18 of 22 human cancer types analyzed (Figure 3A). Expression of *FAM83D* is significantly higher in tumors with a gain of *FAM83D* in comparison to those without such changes, suggesting that the gain of *FAM83D* results in elevated expression of *FAM83D* (Figure 3B, Supplementary Table S2). In a survey of the data available for BC, lung adenocarcinomas and squamous cell carcinomas, ovarian serous cystadenocarcinoma, head and neck squamous cell carcinoma, and sarcoma, more than 10 cases in which there was a loss of *FAM83D* were identified (Supplementary Table S2). In lung squamous cell carcinoma and ovarian serous cystadenocarcinoma, *FAM83D* loss was found to be associated with a significant reduction in *FAM83D* expression (Figure 3B, Supplementary Table S2). Notably, in the case of BC, *FAM83D* was expressed at significantly higher levels in cases with *FAM83D* loss (Figure 3B, Supplementary Table S2), suggesting that additional mechanisms may lead to elevated expression of *FAM83D*.

**Figure 4:**
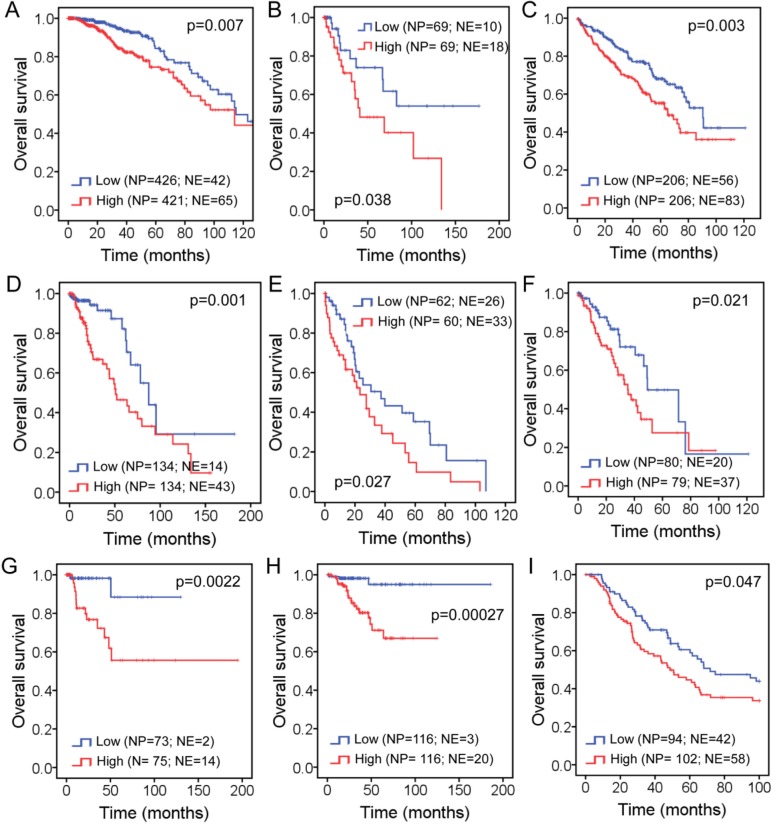
Impact of *FAM83D* expression level on overall survival in patients with: A. Breast cancer, B. Cervical squamous cell carcinoma, C. Kidney renal clear cell carcinoma, D. Brain lower grade glioma, E. Liver hepatocellular carcinoma, F. Lung adenocarcinoma, G. Kidney renal papillary cell carcinoma, H. Uterine corpus endometrioid carcinoma, and I. Skin cutaneous melanoma “NP” refers to “Number of Patients”, whereas “NE” refers to “Number of Events”. Kaplan-Meier estimates of overall survival according to the *FAM83D* expression are presented. The *p* values were obtained from a log-rank test among two groups.

Kaplan-Meier analyses of the TCGA cohort were used to assess the prognostic value of *FAM83D* expression in different human cancer types. We found that BC patients with higher levels of *FAM83D* mRNA have significantly shorter overall survival (Figure 4A), which is consistent with the results of a meta-analysis of microarray datasets. In addition, we discovered that higher levels of *FAM83D* mRNA are also correlated with shorter overall survival in 8 of 16 other human cancer types (Figure 4B to 4I, Supplementary Figure S3). Moreover, it appears that the cervical (Figure 4B), kidney (Figure 4G) and uterine (Figure 4H) carcinomas show a larger difference in overall survival between high and low levels of FAM83D expression in comparison to other types of cancer. Taken together with the results from earlier studies of breast [4] and liver [5] cancers, these latest findings strengthen the proposal that *FAM83D* is a general oncogene in many different human cancer types.

### Mutation of *TP53* is coincident with increased *FAM83D* expression in many human cancer types

**Figure 5:**
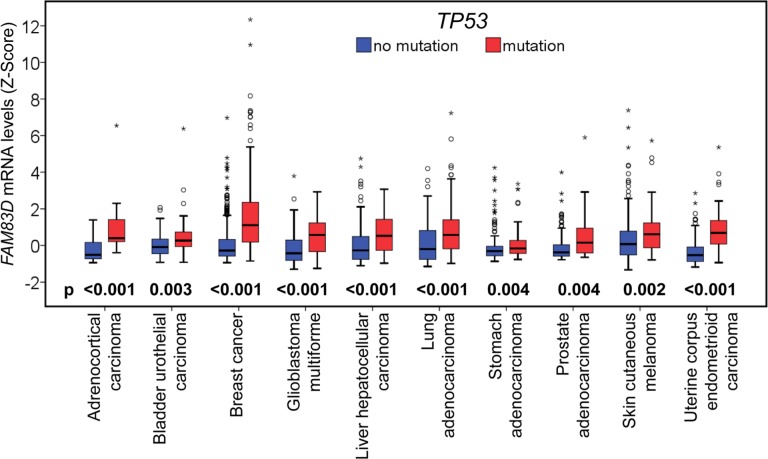
Relationship between *FAM83D* mRNA expression and *TP53* mutations *FAM83D* mRNA expression levels were compared between samples with *TP53* wildtype (no mutation) and mutant forms. The *p*-values were obtained from Mann-Whitney U test. “^O^” signifies an outlier; and “*” an extreme outlier.

As *TP53* is situated at the crossroads of a network of signaling pathways that suppresses cancer development, we sought to investigate the potential relationship between *TP53* and *FAM83D*. 16 of 22 cancer types were used to compare *FAM83D* expression in *TP53* wild-type and mutated cases (Supplementary Table S3). In 10 of 16 cancer types, *TP53* mutations coincided with a significant increase in *FAM83D* expression (Figure 5, Supplementary Table S3), suggesting that *TP53* may be a factor in the regulation of *FAM83D* expression.

### *FAM83D* expression correlates with the level of genomic instability in many human cancer types

FAM83D interacts with the chromokinesin KID22 and is required for correct chromosome congression in metaphase. We therefore investigated whether higher levels of *FAM83D* expression is correlated with the extent of genome instability in cancer samples. TCGA datasets were used to compare *FAM83D* expression to the fraction of cancer genomes with copy number alteration (CNA) and mutation frequencies (Table 2). For 8 cancer types, including breast, low-grade brain gliomas and lung adenocarcinomas, we detected a significant positive correlation between both CNA fractions and mutation frequencies, and *FAM83D* expression level (Spearman's rho, *p* < 0.05) (Table 2). For some other cancers, such as colorectal carcinoma, kidney renal papillary cell carcinoma, pancreatic and prostate adenocarcinoma, a significant correlation was only detected between *FAM83D* expression and either CNA fraction or mutation frequency (Table 2). No correlation was detected for cancers, such as kidney renal clear cell carcinoma, cervical, stomach adenocarcinoma, and ovarian serous cystadenocarcinoma (Table 2). Overall, we conclude that there is a significant correlation between *FAM83D* expression and the level of genome instability in multiple cancer types in the TCGA datasets.

**Table 2:** Association of *FAM83D* expression level with the fraction of cancer genomes with copy number alteration and mutation frequencies (number of mutated genes in each sample)

Cancer types	Mutation frequency		Fraction of copy number altered genome	
	Spearman's rho	*p*-value	Spearman's rho	*p*-value
Adrenocortical Carcinoma	**0.444**	**6.63E-05**	0.223	0.055
Brain Lower Grade Glioma	**0.197**	**0.00098**	**0.391**	**6.74E-12**
Bladder Urothelial Carcinoma	0.129	0.15	0.035	0.7
Breast Cancer	**0.413**	**1.19E-40**	**0.56**	**2.39E-80**
Cervical Squamous Cell Carcinoma	0.114	0.12	**0.167**	**0.022**
Colorectal Adenocarcinoma	−0.071	0.34	**0.166**	**0.026**
Glioblastoma Multiforme	0.013	0.88	**0.237**	**0.0055**
Kidney Renal Clear Cell Carcinoma	−0.096	0.051	−0.049	0.32
Kidney Renal Papillary Cell Carcinoma	0.022	0.78	**0.172**	**0.029**
Liver Hepatocellular Carcinoma	**0.192**	**0.0078**	**0.319**	**7.42E-06**
Lung Adenocarcinoma	**0.336**	**7.88E-06**	**0.259**	**0.00062**
Lung Squamous Cell Carcinoma	0.036	0.63	**0.32**	**0.000013**
Stomach Adenocarcinoma	−0.045	0.53	0.057	0.36
Ovarian Serous Cystadenocarcinoma	0.012	0.88	0.099	0.22
Pancreatic Adenocarcinoma	−0.061	0.59	**0.341**	**0.0016**
Prostate Adenocarcinoma	0.063	0.31	**0.135**	**0.031**
Skin Cutaneous Melanoma	**0.146**	**0.015**	**0.219**	**0.00024**
Head and Neck Squamous Cell Carcinoma	**0.303**	**2.39E-07**	**0.255**	**0.000016**
Sarcoma	NA	NA	**0.419**	**3.04E-12**
Thyroid Carcinoma	−0.00045	0.99	**0.108**	**0.031**
Uterine Corpus Endometrioid Carcinoma	**0.16**	**0.015**	**0.357**	**2.13E-08**
Uterine Carcinosarcoma	**0.294**	**0.028**	0.162	0.23

## DISCUSSION

In this study, we initially performed a meta-analysis of the public microarray profiles to evaluate the prognostic value of *FAM83D* expression in BC. Consistent with the results of the previous study [4], we found that higher levels of *FAM83D* were significantly associated with shorter overall and metastatic relapse-free survival, particularly in patients with ER^+^ and luminal subtype tumors. As no correlation was found in samples from patients with ER^−^ and other subtype tumors, we conclude that the association between *FAM83D* expression and overall and metastatic relapse-free survival in the complete patient sample set, was driven by the ER^+^ and luminal subtype tumors. Using TCGA breast cancer data, we further confirmed that higher levels of *FAM83D* expression significantly reduce overall survival. These results indicate that *FAM83D* is a prognostic biomarker for BC.

Three genetic mechanisms activate oncogenes in human cancers: (1) mutation, (2) gene amplification, and (3) chromosome rearrangements. How is the *FAM83D* oncogene activated? Using TCGA data, we found that *FAM83D* is rarely mutated, but is frequently amplified in the majority of human cancer types. *FAM83D* amplification is strongly correlated with an increase in its expression. In addition, we found that *TP53* mutations coincide with an increase in the expression of *FAM83D*. *TP53* is widely mutated in various human cancers. Therefore, not surprisingly, it has been reported that *FAM83D* expression is elevated in various cancers [18]. Moreover, higher levels of *FAM83D* expression positively correlate with a poor prognosis in many cancer types, including liver hepatocellular carcinoma. Two recent studies have identified *FAM83D* as a prognostic marker for hepatocellular carcinoma [5, 6].

FAM83D was first identified as a spindle protein localizing with the spindle apparatus during mitosis [15]. FAM83D interacts with chromokinesin KID22 and is required for correct chromosome congression during metaphase [15]. The mitotic spindle is responsible for the accurate distribution of sister chromatids during cell division. Functional aberration of the mitotic spindle can lead to errors in chromosome separation and subsequent aneuploidy as often seen in advanced human cancers. In this study, we have found that high levels of *FAM83D* expression are strongly correlated with an increase in genomic instability in the cells of multiple cancer types (Table 2). The findings reported here identify *FAM83D* as a potential oncogene for many human cancer types, and highlight the prognostic value of *FAM83D* expression in cancer outcomes. Further studies, however, will be needed to develop a deeper understanding of the mechanistic roles of *FAM83D* in the development and progression of cancer.

## MATERIALS AND METHODS

### Datasets used in this study

*FAM83D* genomic alterations and mRNA expression levels, *TP53* mutations, fraction of copy number alteration, frequency of gene mutations and clinical information for the set of samples in each TCGA study were obtained from cBioPortal [19, 20]. All samples associated with the datasets analyzed have been included in this study. Further details can be found in Supplementary Tables S1, S2 and S3. Clinical information concerning these samples can be downloaded from cBioPortal (http://www.cbioportal.org/data_sets.jsp).

### Statistical analysis

We performed meta-analysis for breast cancer AE-survival, MR-free survival, breast cancer subtype, and breast cancers with clinicopathological information on 36 breast cancer datasets using bc-GenExMiner v3.1 (the website is: http://bcgenex.centregauducheau.fr/BC-GEM/GEM_Requete.php?mode=1) [16]. The analytical tools available for prognostic gene expression analysis in bc-GenExMiner were used to generate Figure 1 and 2 and Table 1, which include (1) Targeted analysis with N and ER subtyping; (2) Exhaustive analysis with N and ER subtyping; and (3) Analysis by molecular subtype. The difference in *FAM83D* mRNA expression levels between different statuses of *FAM83D* genomic alteration and *TP53* mutation was analyzed by Mann-Whitney U. Kaplan-Meier plots were constructed and a log-rank test was used to determine differences among overall survival according to *FAM83D* mRNA levels in different cancer types. Spearman correlation was used to assess the association of *FAM83D* mRNA levels with CNA and mutation frequencies. All analyses were performed by SPSS 11.5.0 for Windows. A two-tailed *p*-value of less than 0.05 was considered to indicate statistical significance.

## SUPPLEMENTARY FIGURES AND TABLES


