# Supplementary material for: Prognostic significance of FAM83D gene expression across human cancer types
Source: Oncotarget. 2015 Dec 15;7(3):3332–40. doi: 10.18632/oncotarget.6620 (PMC4823109; doi:10.18632/oncotarget.6620)
Supplement: Supplementary file 1 [file oncotarget-07-3332-s001.pdf]

SUPPLEMENTARY FIGURES AND TABLES

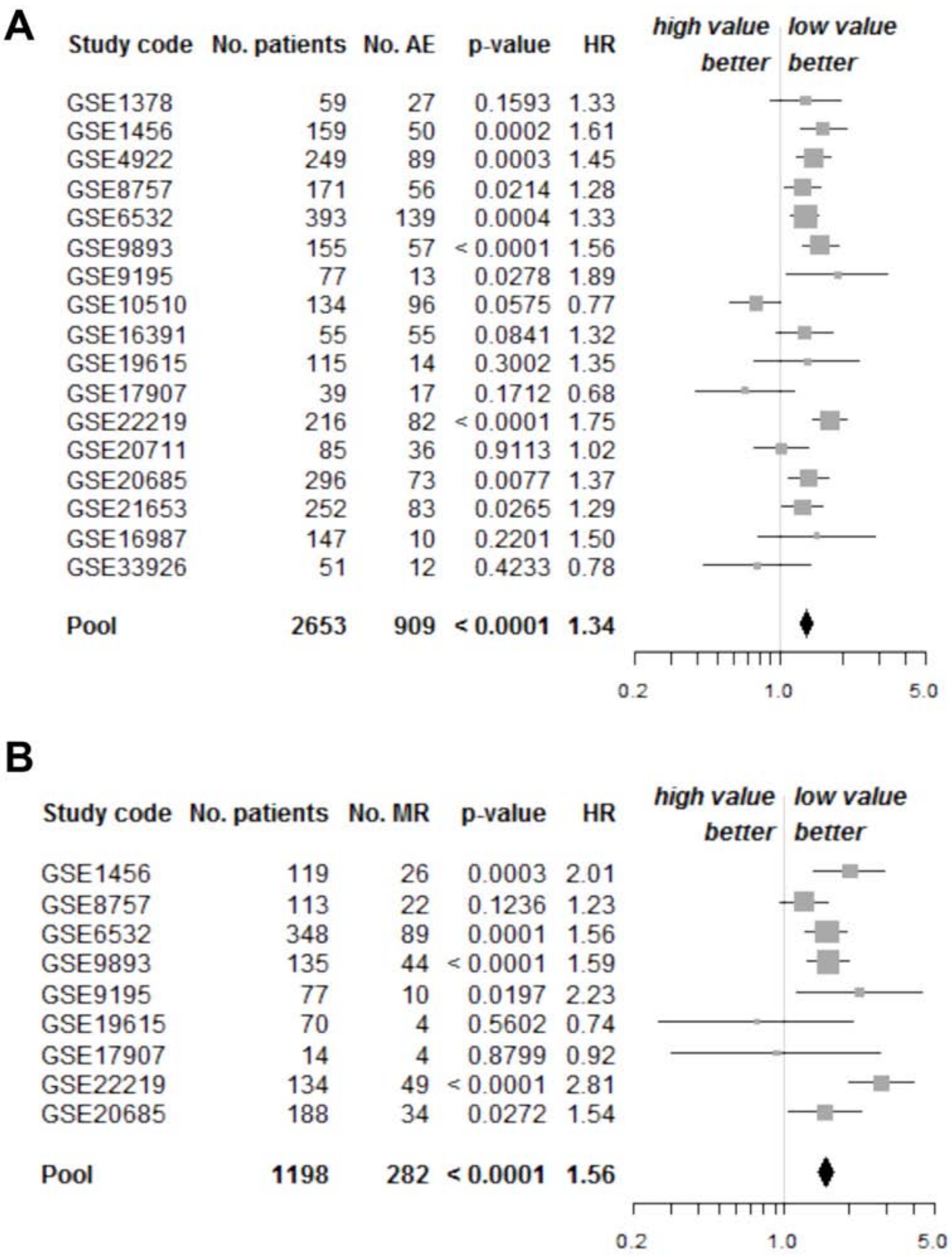

Supplementary Figure S1: Forest plot shows impact of *FAM83D* expression on: A. AE- and B. MR-free survival.

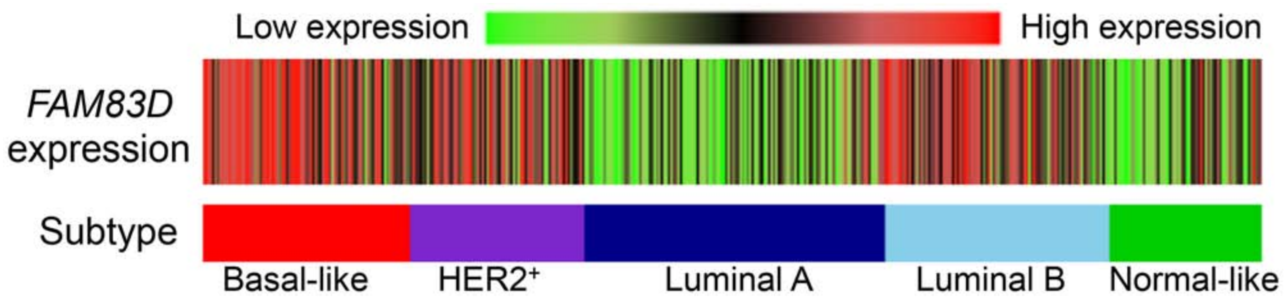

Supplementary Figure S2: *FAM83D* expression in different molecular subtypes.

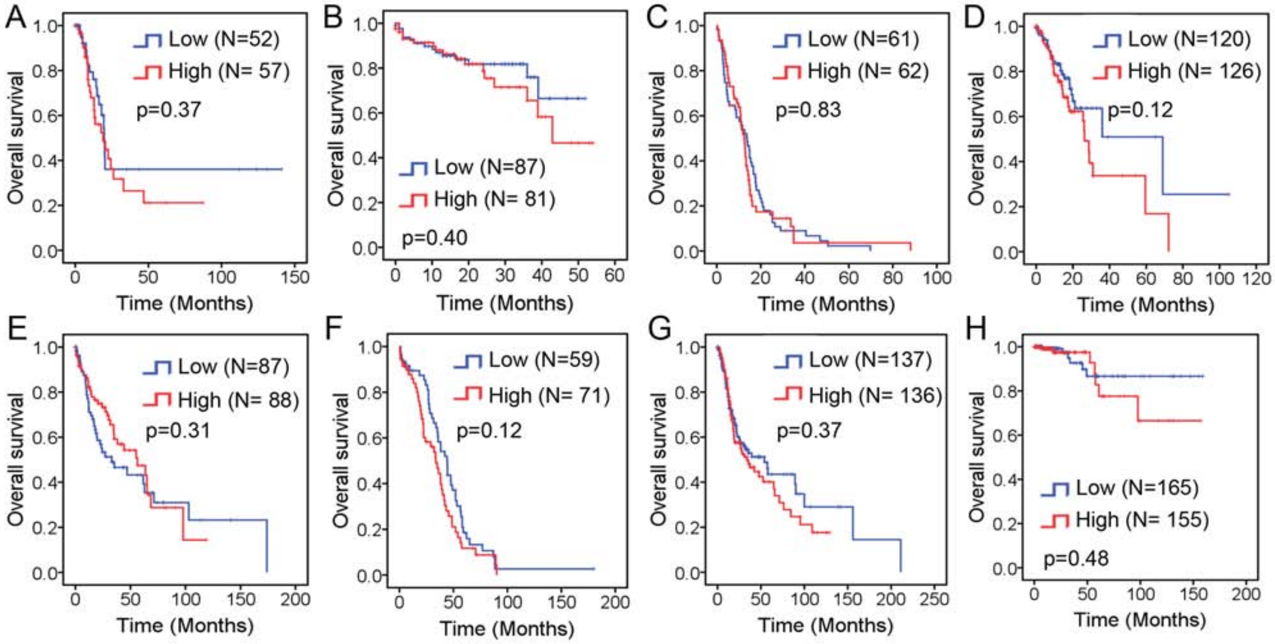

Supplementary Figure S3: Impact of *FAM83D* expression level on overall survival in patients with: A. Bladder urothelial carcinoma, B. Colorectal adenocarcinoma, C. Glioblastoma multiforme, D. Stomach adenocarcinoma, E. Lung squamous cell carcinoma, F. Ovarian serous cystadenocarcinoma, G. Head and neck squamous cell carcinoma, and H. Thyroid carcinoma.

Supplementary Table S1: Information of TCGA datasets used in this study.

Cancer types	Number of patients	Overall survival
Adrenocortical Carcinoma	75	No
Brain Lower Grade Glioma	286	Yes
Bladder Urothelial Carcinoma	126	Yes
Breast Cancer	959	Yes
Cervical Squamous Cell Carcinoma	190	Yes
Colorectal Adenocarcinoma	182	Yes
Glioblastoma Multiforme	136	Yes
Kidney Renal Clear Cell Carcinoma	413	Yes
Kidney Renal Papillary Cell Carcinoma	161	Yes
Liver Hepatocellular Carcinoma	190	Yes
Lung Adenocarcinoma	171	Yes
Lung Squamous Cell Carcinoma	178	Yes
Stomach Adenocarcinoma	258	Yes
Ovarian Serous Cystadenocarcinoma	158	Yes
Pancreatic Adenocarcinoma	83	No
Prostate Adenocarcinoma	257	No
Skin Cutaneous Melanoma	278	Yes
Head and Neck Squamous Cell Carcinoma	279	Yes
Sarcoma	253	No
Thyroid Carcinoma	397	Yes
Uterine Corpus Endometrioid Carcinoma	232	Yes
Uterine Carcinosarcoma	56	No

Supplementary Table S2: *FAM83D* expression in the patients with different copy number changes of *FAM83D*. The *p*-values was obtained by Mann-Whitney test.

Cancer types	loss	no change	gain	p-value (gain vs no change)	p-value (loss vs no change)*
Adrenocortical Carcinoma	2	31	42	0.35	ND
Brain Lower Grade Glioma	1	259	26	**0.007**	ND
Bladder Urothelial Carcinoma	2	38	86	0.059	ND
Breast Cancer	56	500	403	**<0.001**	**<0.001**
Cervical Squamous Cell Carcinoma	5	108	77	**0.010**	ND
Colorectal Adenocarcinoma		46	136	**0.004**	ND
Glioblastoma Multiforme	3	82	51	0.27	ND
Kidney Renal Clear Cell Carcinoma		314	99	**<0.001**	ND
Kidney Renal Papillary Cell Carcinoma	1	105	55	**0.049**	ND
Liver Hepatocellular Carcinoma	6	121	63	**<0.001**	ND
Lung Adenocarcinoma	30	67	74	**<0.001**	0.50
Lung Squamous Cell Carcinoma	18	67	93	0.68	**0.001**
Stomach Adenocarcinoma	1	98	159	**0.008**	ND
Ovarian Serous Cystadenocarcinoma	12	66	80	0.14	**<0.001**
Pancreatic Adenocarcinoma	2	63	18	**0.001**	ND
Prostate Adenocarcinoma	7	241	9	0.36	ND
Skin Cutaneous Melanoma	4	134	140	**<0.001**	ND
Head and Neck Squamous Cell Carcinoma	17	148	114	**0.001**	0.072
Sarcoma	20	134	99	**<0.001**	0.29
Thyroid Carcinoma		386	11	**0.014**	ND
Uterine Corpus Endometrioid Carcinoma	2	203	27	**<0.001**	ND
Uterine Carcinosarcoma	3	10	43	0.72	ND

* ND = not done, because there are no cases or a small number (<10) of cases with loss

Supplementary Table S3: *FAM83D* expression in the patients with *TP53* wild-type or mutations. The *p*-values was obtained by Mann-Whitney test.

**Cancer types**	wild type	mutation	p-value*
Adrenocortical Carcinoma	60	15	**<0.001**
Brain Lower Grade Glioma	139	147	0.16
Bladder Urothelial Carcinoma	63	63	**0.003**
Breast Cancer	664	295	**<0.001**
Cervical Squamous Cell Carcinoma	181	9	ND
Colorectal Adenocarcinoma	89	93	0.17
Glioblastoma Multiforme	93	43	**<0.001**
Kidney Renal Clear Cell Carcinoma	407	6	ND
Kidney Renal Papillary Cell Carcinor	157	4	ND
Liver Hepatocellular Carcinoma	130	60	**<0.001**
Lung Adenocarcinoma	85	86	**<0.001**
Lung Squamous Cell Carcinoma	37	141	0.094
Stomach Adenocarcinoma	141	117	**0.004**
Ovarian Serous Cystadenocarcinom	18	140	0.67
Pancreatic Adenocarcinoma	29	54	0.59
Prostate Adenocarcinoma	233	24	**0.004**
Skin Cutaneous Melanoma	232	46	**0.002**
Head and Neck Squamous Cell Carc	75	204	0.37
Sarcoma	NA	NA	NA
Thyroid Carcinoma	394	3	ND
Uterine Corpus Endometrioid Carcin	165	67	**<0.001**
Uterine Carcinosarcoma	5	51	ND

* ND = not done, because there are a small number (<10) of cases with *p53* mutation. NA = data not available.
